# Real world telehealth delivery of an evidence based self-management education program for people with epilepsy and cognitive comorbidity

**DOI:** 10.3389/fneur.2025.1617539

**Published:** 2025-07-04

**Authors:** Elaine T. Kiriakopoulos, Wren M. Kaden, Lisa P. Sackett, Maureen T. Quigley, Trina Dawson, Laura DeMuro, Kathryn E. Giordano, Jessica DeNaples, Meredith E. Olenec, Stephanie Jennings, Ambereen Burhanuddin, Joanne Harris, Suzanne Lenz, Robert Ross-Shannon, Todd MacKenzie, Barbara Jobst

**Affiliations:** 1 Geisel School of Medicine, Dartmouth College, Hanover, NH, United States; 2 Department of Neurology, Dartmouth Health, Lebanon, NH, United States; 3 The Dartmouth Institute for Health Policy and Clinical Practice, Dartmouth College, Lebanon, NH, United States; 4 Department of Biostatistics, Dartmouth College, Hanover, NH, United States

**Keywords:** epilepsy self-management, quality of life, cognitive function, translation, cognitive coaching, telehealth delivery

## Abstract

**Introduction:**

Cognitive dysfunction is prevalent in epilepsy, and is associated with decreased quality of life. HOme Based Self-management and COgnitive Training CHanges lives (HOBSCOTCH) is an evidence-based self-management program designed for people with epilepsy and comorbid subjective cognitive dysfunction. This project examines the delivery of HOBSCOTCH outside of the research setting as it translates from science to service.

**Methods:**

People with epilepsy and subjective cognitive dysfunction (*n* = 205) enrolled in the HOBSCOTCH program over an 11-month period (3/11/24–2/12/25) and completed pre-and post-self-report assessments in which demographics, perceived cognition (Everyday Memory Questionnaire-Revised), quality of life (Quality of Life in Epilepsy-10), shared decision-making (CollaboRATE), and program satisfaction were measured.

**Results:**

After completion of the 8-week HOBSCOTCH education program with a Cognitive Coach, participant quality of life and subjective cognition both significantly improved (*p* < 0.001). Program satisfaction was high, as was shared decision-making.

**Discussion:**

HOBSCOTCH Cognitive Coaches deliver a participant-driven one-on-one education program by telehealth, which empowers people with epilepsy to manage their disease and related cognitive symptoms to improve quality of life. Ensuring this low risk behavioral intervention addressing cognitive challenges and quality of life is broadly available is paramount to improving meaningful supports for people with epilepsy.

## 1 Introduction

In the United States, 3.4 million people live with active epilepsy (1). Even when seizures are well controlled, challenges often persist leading to increased morbidity and mortality, and decreased quality of life (QOL) (2–4). Cognitive dysfunction, encompassing difficulties with memory, attention and learning, represents one key comorbid challenge for people with epilepsy (PWE) (5–9). As many as 80% PWE experience cognitive dysfunction, making this comorbidity one which requires innovative and accessible solutions to enable patients to feel more in control of their daily lives (5, 10–13).

Epilepsy, controlled or uncontrolled, is frequently accompanied by persistent cognitive side effects (5, 10, 14). Although seizures are the most prominent feature of epilepsy, many patients describe day to day cognitive dysfunction as a priority on their list of challenges related to epilepsy and the treatments for epilepsy (15–17). The cognitive dysfunction reported in epilepsy covers a spectrum of cognitive domains including short term, spatial and working memory (18, 19), verbal learning, retrieval, attention, planning and psychomotor speed (20). Deficits in language, particularly around decreased naming and fluency, are also commonly reported (14, 21, 22). Although the impactful consequences of cognitive dysfunction in epilepsy is well recognized, treatment options, rehabilitation programs and management strategies for PWE to address cognitive challenges have been insufficient (23).

The need for cognitive rehabilitation is supported by studies revealing cognitive function as a significant predictor of self-evaluation of quality of life in people with epilepsy (24, 25). A recent epidemiologic study from the Centers for Disease Control and Prevention shares data from the 2021 and 2022 adult National Health Interview Survey Sample (*n* = 57,088) that revealed compared with adults with no epilepsy, the 1.2% of US adults with active epilepsy were more likely to have 4 or more co-occurring chronic conditions (26). The most prevalent non psychiatric comorbidity identified for people with active epilepsy in this sample was difficulty remembering (55.8%) (26). The largest absolute differences between those with active versus no epilepsy were difficulty remembering (55.8% vs. 19.1%); and compared with adults with inactive epilepsy, adults with active epilepsy had higher percentages difficulty remembering (55.8% vs. 40.6%). (16).

Epilepsy self-management (ESM) is an evidence-based approach that helps PWE learn skills to better manage their epilepsy and its effect on daily life (27). Broad areas targeted by ESM include treatment adherence, seizure management, coping with comorbid cognitive dysfunction and mood disorders, and lifestyle management. Evidence for the benefits of ESM programs has grown over the past decade, with fifteen randomized controlled trials supporting the benefits of self-management interventions on QOL and related outcomes (28, 29). Despite this evidence, however, ESM has been challenging to bring to clinical centers and to sustain in community service organization settings. This highlights the need for ESM programming with an infrastructure that supports real world access and delivery outside of clinical trials and for there to be collection of pragmatic and relevant real world implementation and outcome data.

Progress has been made with the establishment of a centralized support for the HOBSCOTCH (HOme Based Self-Management and COgnitive Training CHanges Lives) ESM program (29–33). Dartmouth Health’s HOBSCOTCH Institute for Cognitive Health and Well-Being^1^ (HI) was founded to traverse barriers and facilitate adoption of and referral to the HOBSCOTCH program. HI has four foundational pillars: Education, Research, Training, and Partnering to expand self-management program access and growth. The Institute engages diverse collaborators in academia, healthcare, industry, government, and the community to maximize equitable impact for PWE and cognitive dysfunction.

The current program evaluation examines translation of a telehealth accessible evidence-based ESM program, HOBSCOTCH, from science to service to meet the needs of PWE.

## 2 Materials and methods

### 2.1 Epilepsy self-management intervention

HOBSCOTCH is an evidence-based program which targets cognitive dysfunction in adults with epilepsy (30–33). The program is delivered by a trained HOBSCOTCH Cognitive Coach one-to one over 8 sessions (1 h per week) by telehealth, incorporating online and telephone components. It is designed to assist PWE in managing and coping with cognitive problems, to help participants lead happier and more productive lives. The HOBSCOTCH intervention represents an innovative way of addressing neurocognitive difficulties and improving QOL in PWE. The program fosters an understanding of foundational epilepsy and cognition concepts, and aids patients in building skills in self-awareness and mindfulness to help them identify situations and conditions under which their cognitive problems tend to occur (Table 1). The intervention then proceeds to help PWE build new cognitive skills through Problem Solving Therapy and the implementation of compensatory memory strategies. This framework empowers PWE to feel more in control of their challenges as they drive the innovative solutions applied to their self-identified problems throughout the program.

**Table 1:** HOBSCOTCH intervention components.

Component description	HOBSCOTCH session
Rapport building with cognitive coach	Presession
Education module	Session 1
Self-awareness training exercises	Session 1 & 2
Problem solving therapy	Sessions 2–8
Compensatory memory strategies	Sessions 1–8
Mindfulness exercises	Sessions 1–8
Maintenance planning	Session 8

### 2.2 HOBSCOTCH institute team

The HOBSCOTCH Institute (HI) team is a multidisciplinary team that includes physicians, public health educators, nurses, psychologists, and community health workers. The HI team is trained and skilled to work with PWE and their families both in adjunct care, education and research environments and roles. At the time of data collection, Cognitive Coaches n = 17; coaches vary in gender (male = 1, female = 15, other = 1, prefer not to answer = 1), age (18–24 = 3, 25–34 = 4, 35–44 = 3, 45–54 = 3, 55–64 = 1, 65 + = 2), race (White = 14, Asian/Pacific Islander = 2), ethnicity (Hispanic or Latino = 1, not Hispanic or Latino = 15), and highest degree held (high school = 1, bachelor’s degree = 5, master’s degree = 7, doctoral degree = 3). Cognitive Coach training consists of 8-h of virtual interactive training that includes didactic teaching with formal discussion and question and answer periods, case presentations and modeling of coach-participant sessions, simulated patient problem solving by trainees guided by Master Cognitive Coaches (34).

### 2.3 Participant enrollment pathways

National access to the HOBSCOTCH program is available to PWE through multiple sources, including HIPAA compliant clinician or community service organization referral to the program and self-referral via online registration through the Dartmouth Health website^2^ or the Centers for Disease Control and Prevention Managing Epilepsy Well website.^3^

Participants who register for the program are prescreened by a program coordinator at the HI to ensure they are familiar with the program components and the commitment of 8 one-hour sessions with a Cognitive Coach delivered by telehealth over an 8-week time period. Following the coordinator prescreen, participants are matched with a certified Cognitive Coach and an initial rapport building telehealth call (phone or online) is completed so that the participant and the Coach can familiarize themselves with one another and determine a weekly schedule for meeting to complete the HOBSCOTCH sessions (Table 1).

### 2.4 Demographic variables

Data elements included were sociodemographic variables of age, gender (Man, Woman, Other, Unspecified, Prefer not to answer), race (White, Black or African American, Asian, American Indian or Alaskan Native, Native Hawaiian or Pacific Islander, Other, Prefer not to answer), ethnicity (Hispanic or Latino, not Hispanic or Latino, Prefer not to answer), occupation (Employed full time, Employed part time, Unemployed and currently looking, Unemployed and not currently looking, Student, Retired, Homemaker, Self-employed, Unable to work, Prefer not to answer), marital status [Single (never married), Married or in a domestic partnership, Member of an unmarried couple, Divorced, Widowed, Separated, Prefer not to answer], and education (Less than a high school diploma, High school diploma or equivalent, Some college no degree, Associate’s degree, Bachelor’s degree, Master’s degree, Professional degree, Doctorate).

### 2.5 Education program evaluation

Participants (*n* = 205) registered for the HOBSCOTCH telehealth delivered education program during an 11-month period from (3/11/24–2/12/25) and completed screening with a program coordinator, voluntary demographic questionnaires and program evaluation and satisfaction surveys (Table 2). Screening data collected included geographic data, self-reported seizure control, and past participation in cognitive rehabilitation, mindfulness and self-management programs.

**Table 2:** HOBSCOTCH program evaluation variables and timepoint(s) collected.

Outcome	Program pre-screen	Pre-HOBSCOTCH questionnaire	Post-HOBSCOTCH questionnaire	Information source
Referral Source	**X**			Clinician, community organization, PWE
Demographics		**X**		PWE
Severity of Epilepsy (GASE)	**X**			PWE
Past 30-day seizure	**X**			PWE
Past 12-month seizure	**X**			PWE
Prior experience with ESM	**X**			PWE
Quality of Life (QOLIE-10)		**X**	**X**	PWE
Subjective Cognition (EMQ-R)		**X**	**X**	PWE
Shared decision making (collaboRATE)			**X**	PWE
Acceptability, Program (8 Session) Completion				Cognitive Coach
Satisfaction			**X**	PWE

GASE = Global Assessment Severity of Epilepsy; PWE = Person with Epilepsy; QOLIE-10 = 10 Item Quality of Life in Epilepsy Scale; Subjective Cognition Measure EMQ-R = Everyday Memory Questionnaire; collaboRATE = 3 item shared decision-making measure. X indicates the data collection timepoint (s).

### 2.6 Program evaluation measures

#### 2.6.1 Global assessment of severity of epilepsy (GASE)

The GASE is a one-item Likert-type measure assessing patient-perceived severity of epilepsy from not at all severe to very severe (35). PWE with higher GASE scores are less likely to achieve 1-year seizure freedom and more likely to be on more antiseizure medications, experience more side effects from medication, endorse more depression and anxiety symptoms, and have increased self-reported seizure-related disability. The identified determinants of global, self-rated epilepsy severity can aid the evaluation of appropriate interventions and support services for PWE (35).

#### 2.6.2 Quality of life in epilepsy-10 (QOLIE-10)

QOL was assessed with the QOLIE-10 instrument, a self-administered questionnaire developed from the original QOLIE-89 (36) with scores ranging from 0.1–5.1. Higher scores indicate worse QOL. The scale comprises seven components, including seizure worry, overall QOL, emotional well-being, energy-fatigue, cognitive functioning, medication effect, and social function. The QOLIE-10 has demonstrated good test–retest reliability and correlates well with longer versions of this instrument (37).

#### 2.6.3 Everyday memory questionnaire (EMQ-R)

The Everyday Memory Questionnaire (EMQ) was developed as a subjective measure of memory failure in everyday life. Reliability and factor analysis of the initially developed EMQ-28 identified two main factors, general memory and attentional function. Further analysis reduced the questionnaire to a 13-item measure (EMQ-R), with two main factors (Retrieval and Attentional tracking), strong internal reliability, and good discriminatory properties between clinical and control groups. The 28-item questionnaire consistently differentiated between two broad systems of memory and attention, with some differentiation of visual and verbal, or language systems. The revised, 13-item questionnaire is a valid and reliable tool that has good face validity for use with neurological patients (38, 39). While comparisons with objective memory tests are varied, the EMQ-R has been shown to be a reliable and valid test of an individual’s beliefs about their memory and its impact on their day-to-day life (38).

#### 2.6.4 CollaboRATE (shared decision-making)

Shared decision-making is the involvement of patients in their own healthcare decisions and is crucial for patient engagement and patient-centered care delivery. The CollaboRATE tool is a brief, 3-question rating scale that was designed to evaluate the patient experience in the shared decision-making process. This patient-centric measure analyzes effort of the healthcare team in three distinct categories: (1) help understanding one’s own health issues, (2) listening to things that matter most to the patient about their own health issues, and (3) including what matters most to the patient in choosing next steps in care delivery. Given that the HOBSCOTCH program is a participant driven program, the CollaboRATE tool was operationalized to help measure coaching fidelity to HOBSCOTCH’s intended participant driven design by asking program participants to rate the quality of their Cognitive Coach’s shared decision-making communication throughout the HOBSCOTCH sessions at a general, high level. collaboRATE has demonstrated discriminative validity, with a significant increase in collaboRATE score as the number of core dimensions of shared decision-making increased from zero (mean score: 46.0, 95% CI 42.4–49.6) to 3 (mean score 85.8, 95% CI 83.2–88.4). collaboRATE also demonstrated concurrent validity with other measures of SDM, excellent intra-rater reliability, and sensitivity to change (40, 41).

### 2.7 Data analysis

Descriptive statistical analysis was conducted using proportions (percentages), means with standard deviation (SD). We tested if there was a change from pre to post in the endpoints using Wilcoxon Signed Rank. Statistical analyses were performed in SPSS (42) and the R programming language (43). Participant addresses were geocoded using ArcGIS Pro v3.3.1 and associated with CDC/ATSDR Social Vulnerability Index (SVI) at the Census tract level.

### 2.8 Ethics review and data collection

All assessments were collected by digital survey using REDCap (Research Electronic Data Capture), a secure web application for building and managing online surveys and databases (42, 43). Evaluation of the general service-oriented delivery of the HOBSCOTCH education program by HOBSCOTCH Institute trained Cognitive Coaches was determined to be exempt from institutional review board (IRB) oversight as a non-human research quality improvement and program evaluation project.

## 3 Results

### 3.1 Sample characteristics

**Figure 1:**
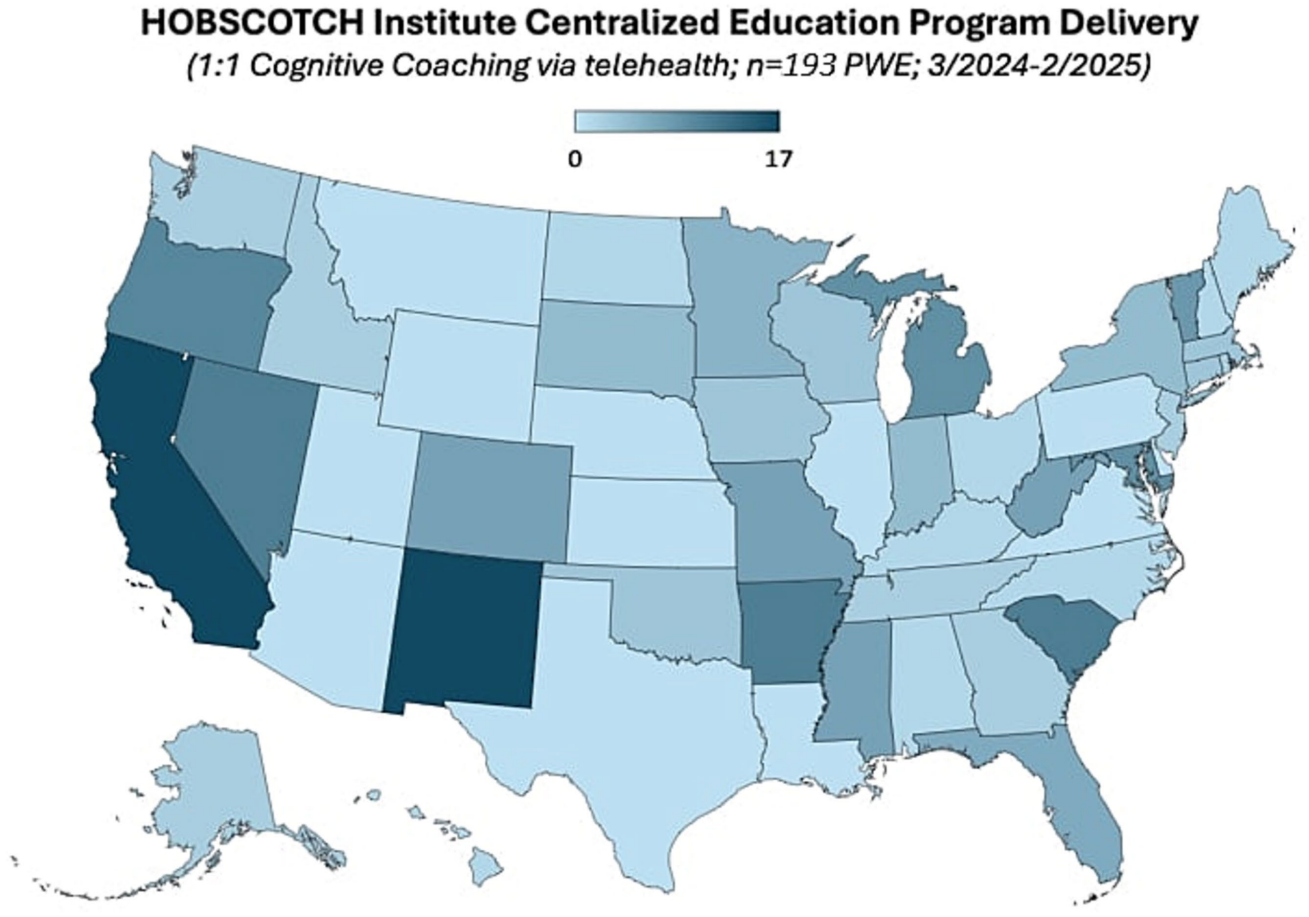
State of residence of people with epilepsy enrolled in the HOBSCOTCH education program (*n* = 191).

**Figure 2:**
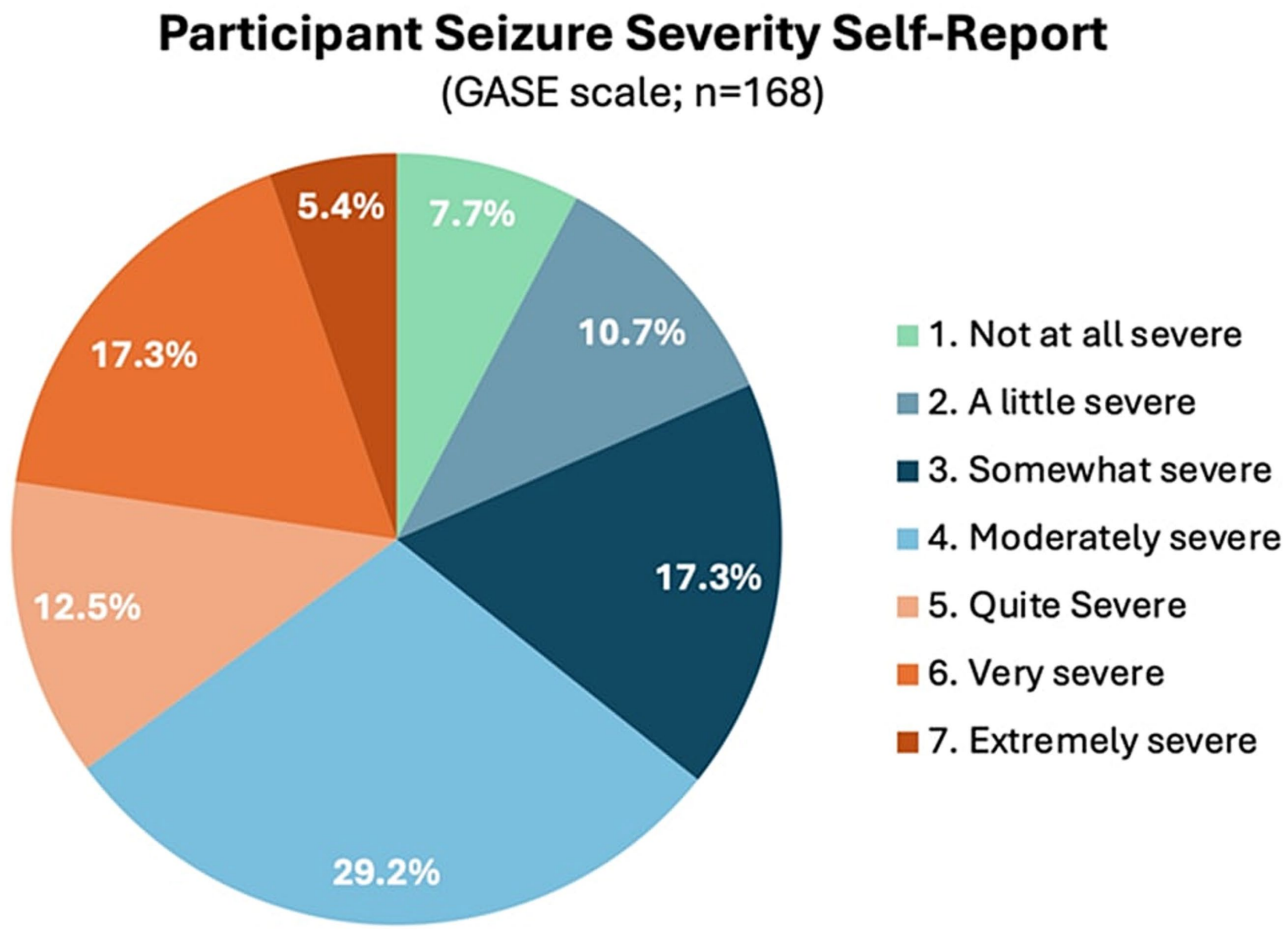
Epilepsy severity as measured by the one-item global assessment of severity of epilepsy (GASE, *n* = 168; *n* = 34 were enrolled prior to implementing this measure).

**Figure 3:**
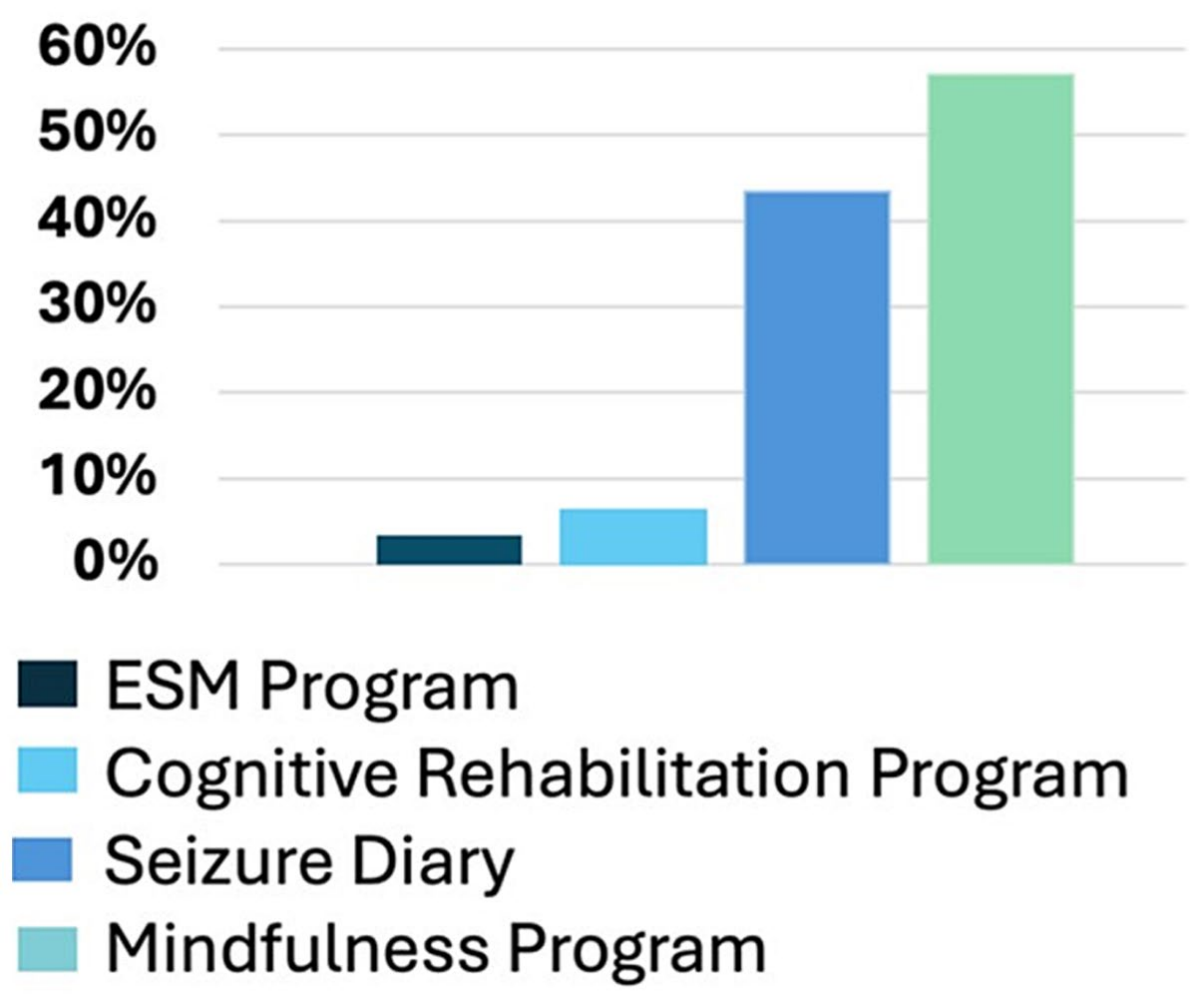
Prior experience with epilepsy self-management, cognitive rehabilitation, seizure diary or tracking, and mindfulness, mediation, or relaxation exercises (*n* = 205).

Participants who participated in prescreening with a HOBSCOTCH program coordinator, were matched with a coach and completed the 8-session program (*n* = 205) reported 38 states of residence (Table 3; Figure 1) and are included in this analysis. Additional participants (*n* = 13; 3.9%) who completed screening process but did not complete the program because of medical reasons or personal/family stressors, and participants who completed some of the program sessions (*n* = 29; 8.9%) are not included in the analysis. Participants who completed the screening process and have been linked to a Cognitive Coach and are actively engaged in program completion (*n* = 75) during the time period are not included in our data analysis. The HOBSCOTCH program was delivered to all participants by telehealth (online, telephone). Each of the 8 sessions was delivered in one-to-one Coaching sessions that lasted approximately one hour. Participants reported a spectrum of epilepsy severity from not at all severe to extremely severe on the GASE scale (*n* = 168) (Table 4; Figure 2). Participant (*n* = 205) experience with, (1) prior ESM was limited to 3% of the sample, (2) a cognitive rehabilitation program (6%), (3) use of a seizure diary (43%) and (4) mindfulness program (57%) (Figure 3).

**Table 3:** Demographic characteristics of people with epilepsy enrolled in the HOBSCOTCH education program (*n* = 205).

Variable	*n*	%
Age (M = 45.38, SD = 15.44, range = 16-83)		
Gender		
Man	70	34%
Woman	132	64%
Other	2	1%
Prefer not to answer	1	0%
Race		
American Indian or Alaskan Native	1	0%
Asian	3	1%
Black or African American	13	6%
Native Hawaiian or Other Pacific Islander	0	0%
White	173	84%
Other	10	5%
Prefer not to answer	5	3%
Ethnicity		
Hispanic or Latino	17	8%
Not Hispanic or Latino	180	88%
Prefer not to answer	8	4%
Occupation		
Employed full time	61	30%
Employed part time	17	8%
Unemployed and currently looking	9	4%
Unemployed and not currently looking	14	7%
Student	11	5%
Retired	29	14%
Homemaker	8	4%
Self-employed	16	8%
Unable to work	38	19%
Prefer not to answer	2	1%
Marital status		
Single (never married)	71	35%
Married or in a domestic partnership	96	47%
Member of an unmarried couple	6	3%
Divorced	26	13%
Widowed	3	1%
Separated	0	0%
Prefer not to answer	3	1%
Education		
Less than a high school diploma	3	1%
High school diploma or equivalent	23	11%
Some college, no degree	43	21%
Associate’s degree	20	10%
Bachelor’s degree	68	33%
Master’s degree	33	16%
Professional degree	10	5%
Doctorate	5	2%

**Table 4:** Participant-reported seizure control in people with epilepsy enrolled in the HOBSCOTCH education program (*n* = 205).

Variable	*n*	%
Controlled seizures		
Yes	93	45%
No	103	50%
Unsure	9	4%
Seizure in past 30 days		
Yes	82	40%
No	96	47%
Not collected	27	13%
Seizure in past 12 months		
Yes	126	61%
No	55	27%
Not collected	23	11%
Self-reported treatment history		
Antiseizure medications	197	96%
Surgery	56	27%
Neurostimulation	19	9%
Medically Rx diet	7	3%

### 3.2 Geographic distribution, social vulnerability index

**Figure 4:**
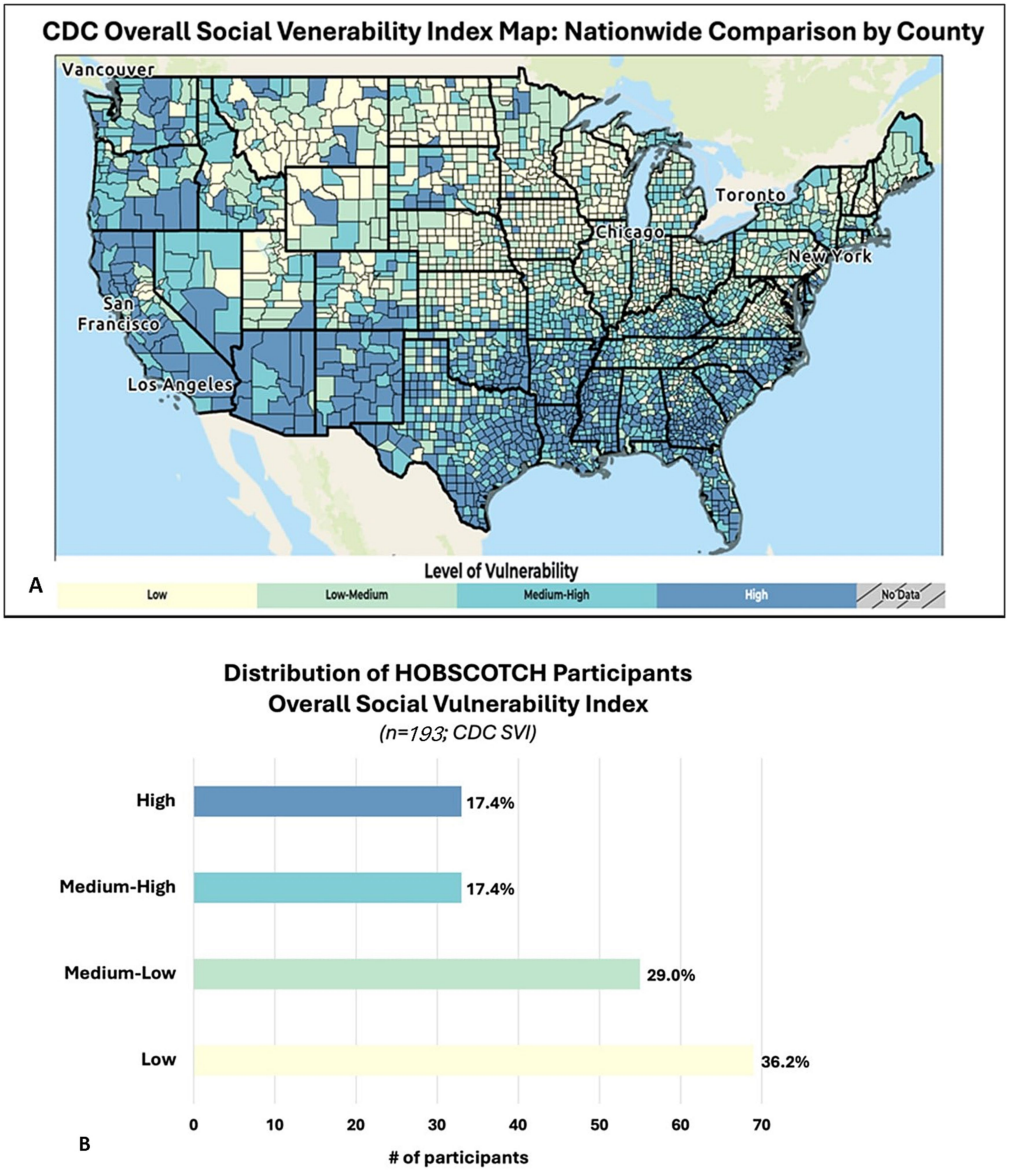
**(A)** Social vulnerability refers to the demographic and socioeconomic factors that contribute to communities being more adversely affected by public health emergencies and other external hazards and stressors that cause disease and injury. Factors such as poverty, lack of access to transportation, and crowded housing may weaken a community’s ability to respond and adapt to stressors and public health emergencies. The CDC/ATSDR Social Vulnerability Index (SVI) is a place-based index, database, and mapping application designed to identify and quantify communities experiencing social vulnerability. **(B)** PWE participating in the telehealth delivered HOBSCOTCH program were geographically distributed across low to high areas based on the SVI maps. Slightly over one third (34.8%) of participants who engaged in the HOBSCOTCH program were located in high and medium-high areas of vulnerability as defined by the overall SVI.

Based on the Federal Office of Rural Health Policy’s rural tract designations, participants resided in rural (15%), not rural (79%) and international locations (6%) (44). Utilizing the Center for Disease Control and Prevention’s Social Vulnerability Index (SVI; Figure 4A) revealed slightly over one third (34.8%) of participants who engaged in the HOBSCOTCH program were located in high and medium-high counties of vulnerability as defined by overall SVI (45, 46) (Figure 4B).

### 3.3 Quality of life and subjective cognition outcomes

Pre- and post-program measures were selected for ongoing program evaluation with specific care to consider participant burden in completing surveys, and to provide the ability to monitor fidelity of program delivery based on expected outcomes established in the two prior HOBSCOTCH randomized controlled trials. The QOLIE-10 and EMQ-R were well received by participants and 95% of participants completed voluntary pre and post surveys.

**Figure 5:**
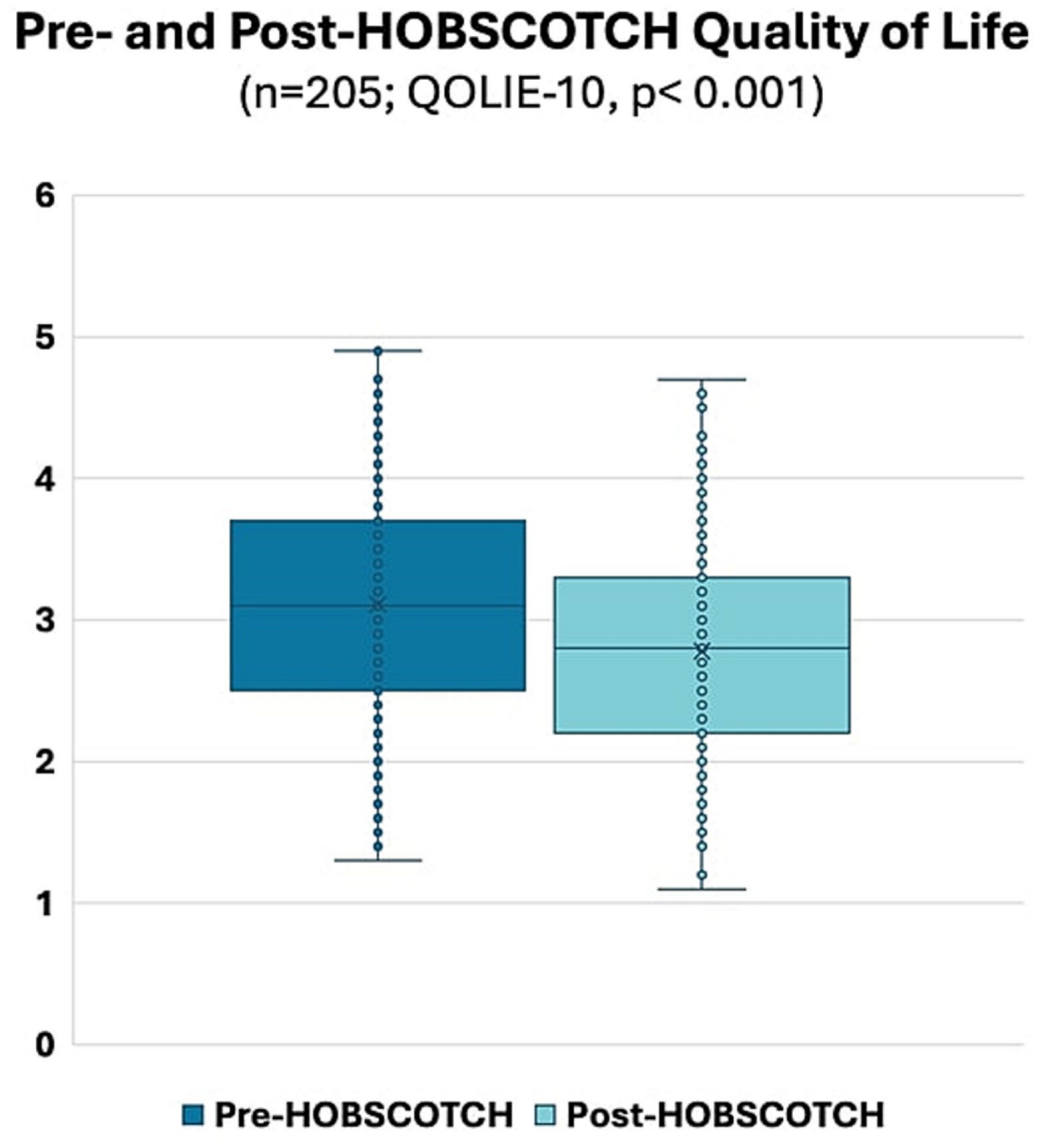
Pre- and post-HOBSCOTCH quality of life as measured by the Quality of Life in Epilepsy-10 (QOLIE-10; *n* = 205); difference assessed using a Wilcoxon Signed Rank test was statistically significant (*p* < 0.001).

**Figure 6:**
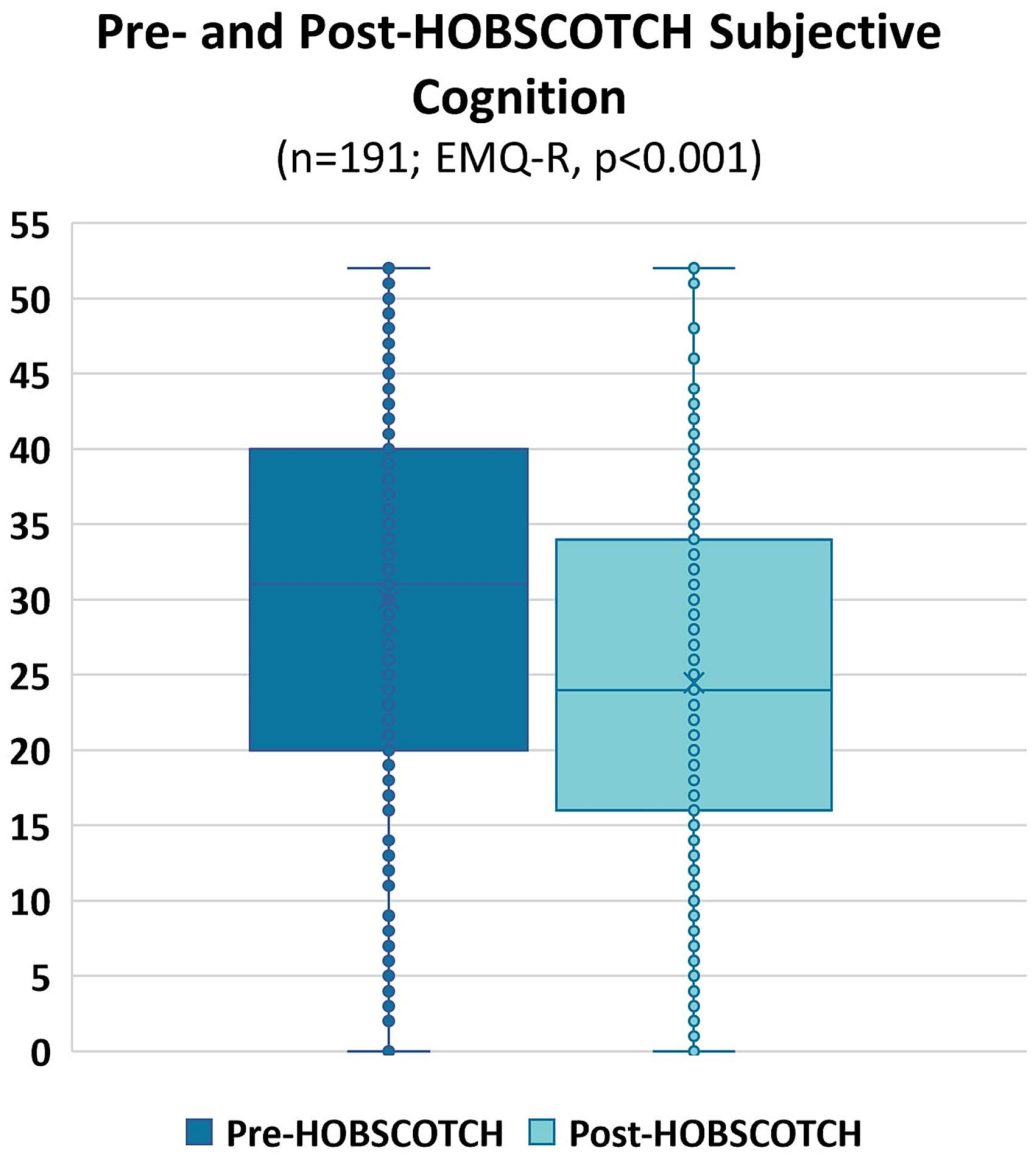
Pre- and post-HOBSCOTCH subjective cognition as measured by the Everyday Memory Questionnaire-Revised (EMQ-R; *n* = 191); difference assessed using a Wilcoxon Signed Rank test was statistically significant (*p* < 0.001).

A Wilcoxon Signed Rank nonparametric test revealed a significant improvement in the QOLIE-10 between pre-HOBSCOTCH (M = 3.11, SD = 0.80, range = 1.3–4.9) and post-HOBSCOTCH (M = 2.78, SD = 0.73, range = 1.1–4.7) assessments (*p* < 0.001, mean change = −0.33, change SD = 0.59; Figure 5). Similarly, a second Wilcoxon Signed Rank nonparametric test revealed a significant improvement in the EMQ-R between pre-HOBSCOTCH (M = 30.01, SD = 12.90, range = 0–52) and post-HOBSCOTCH (M = 24.46, SD = 12.43, range = 0–52) assessments (*p* < 0.001, mean change = −0.33, change SD = 0.59; Figure 6).

### 3.4 Evaluation of shared decision making with cognitive coach

**Figure 7:**
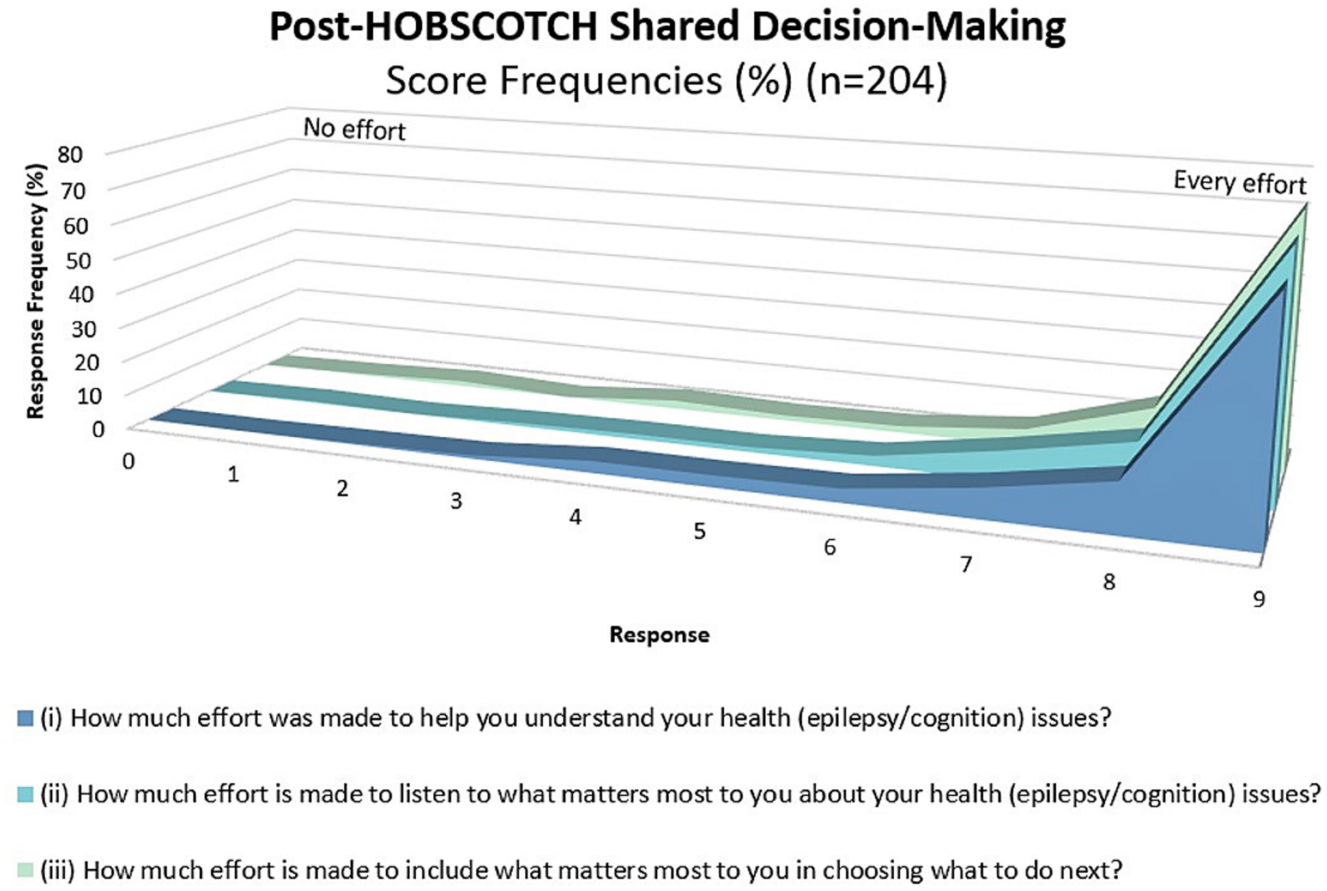
Shared decision making as measured using the collaboRATE during the HOBSCOTCH education program between participant and Cognitive Coach (*n* = 204).

As measured by the collaboRATE, shared decision making between Cognitive Coach and participants (*n* = 204) was high (Figure 7). The majority (87.9%, *n* = 179) ranked their Coach’s effort to help them understand their epilepsy and cognitive difficulties as high (7 or higher on 0–9-point scale). Perception of the Coach’s effort to listen to what is most important to the individual was similarly high at 92.7% (*n* = 189), as was the Coach’s inclusion of what was most important to the participant moving forward (92.1%, *n* = 187).

### 3.5 Satisfaction with the HOBSCOTCH education program

**Figure 8:**
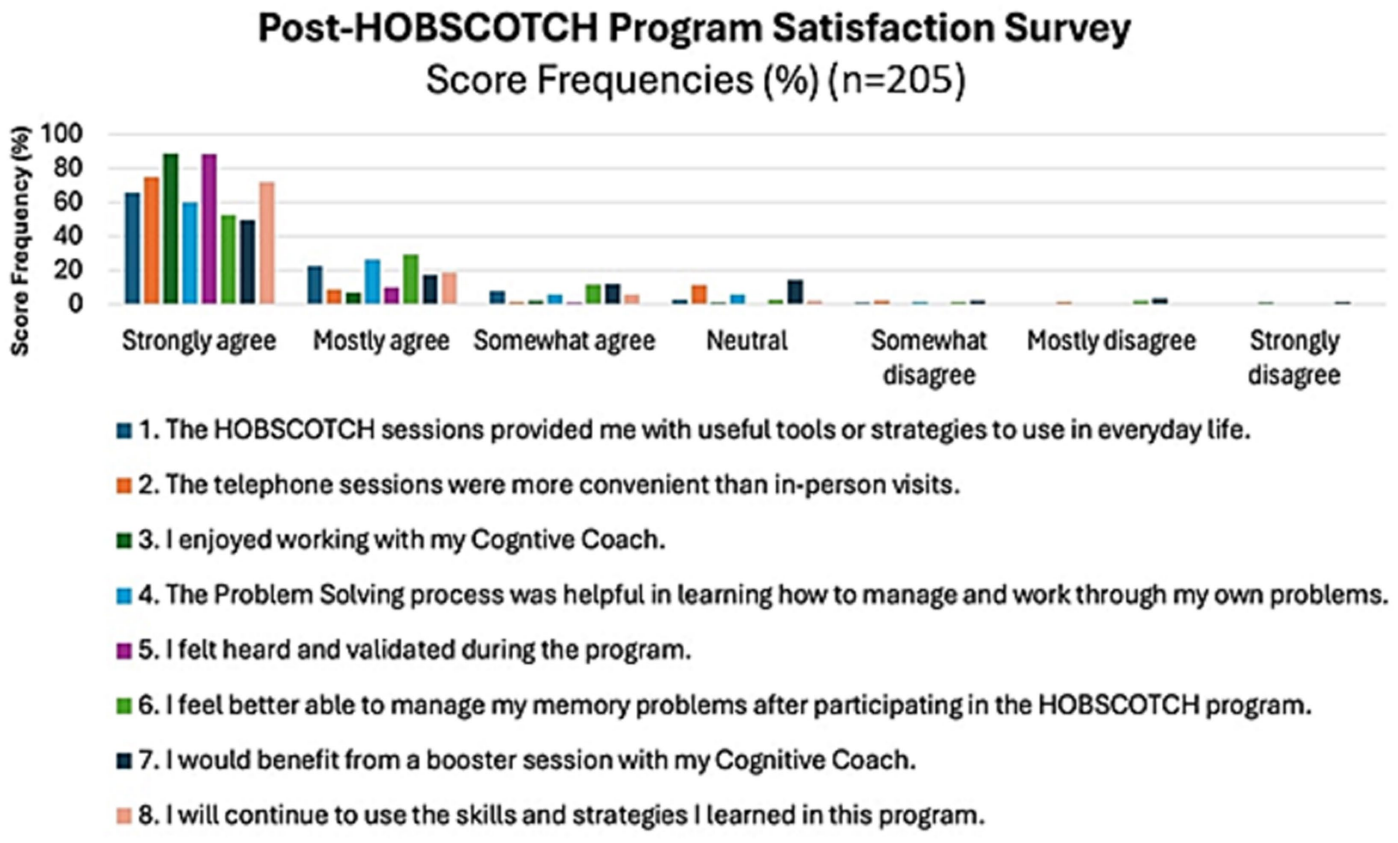
Participant program satisfaction with the HOBSCOTCH intervention (*n* = 205).

**Figure 9:**
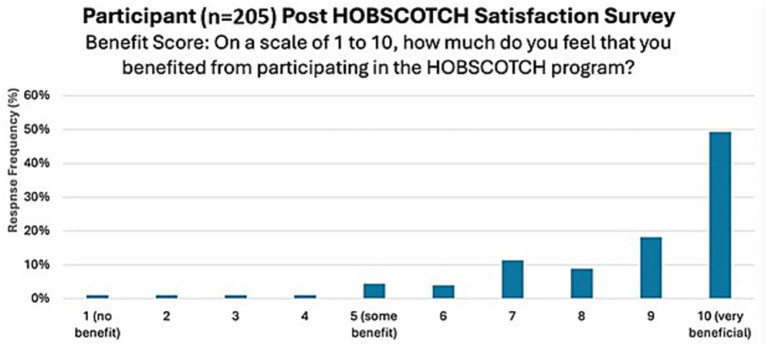
Perceived level of benefit of PWE following their participation in the HOBSCOTCH telehealth education program (*n* = 205).

The majority of PWE, 88.2% (*n* = 180), strongly agreed/agreed that HOBSCOTCH provided them with useful tools or strategies to use in everyday life; the majority, 82% (*n* = 168) strongly agreed/agreed that they felt better able to manage their memory problems after participating in HOBSCOTCH and 90.7% (*n* = 184) strongly agreed or agreed that they would continue to use the skills and strategies learned in the HOBSCOTCH program (Figure 8). Participants overall perceived benefit of HOBSCOTCH was collected as part of the post intervention satisfaction survey, with results indicating 95% (*n* = 192) PWE found engagement with the HOBSCOTCH education program was somewhat beneficial to very beneficial (Figure 9).

## 4 Discussion

A strong base of evidence for the effectiveness of ESM providing significant benefit to PWE has grown over the past decade (27–29). Despite this, translation from science to routine service delivery with ESM as a best practice standard in clinical care streams remains a challenge. This report presents real-world participation by a diverse group of PWE in the telehealth-delivered HOBSCOTCH self-management and cognitive training program. It highlights post-program self-reported outcomes utilizing pragmatic measures, demonstrating HOBSCOTCH’s effectiveness in improving quality of life and subjective cognitive function outside of a randomized controlled clinical trial setting. Data analysis from the National Health Interview Survey (2021,2022) revealed that among adults with active epilepsy, the most prevalent non-psychiatric comorbidity reported by PWE was difficulty remembering (26); supporting the need to make a rigorously tested self-management intervention with a focus on cognition, such as HOSCOTCH, a routinely available support a much needed priority.

The translational effort presented here builds on more than a decade of data highlighting the impact of the Managing Epilepsy Well Network’s self-management interventions on health and quality of life through 15 randomized controlled trials (28). Other examples of pragmatic delivery of psychoeducation in epilepsy include efforts to address comorbid mental health challenges. Psychoeducation support has been accessible in neurology clinics, via support groups, and within structured written materials with beneficial outcomes for PWE (47, 48). Outside of neurologic conditions, self-management education is broadly accessible and integrated into routine diabetes care, with diabetes self-management education having significant clinical benefits on diabetic glycemic control and reduction in the onset of complications (49–52). A recent study has demonstrated that diabetes self-management education is associated with significantly lower spending in total medical and prescription costs for older Medicare beneficiaries (53). Comparable large scale studies in diverse populations of PWE examining health benefits and cost effects of self-management are absent.

Pragmatic measures play a critical role in supporting evidence-based program implementation, addressing stakeholder concerns, and driving quality improvement efforts (54, 55). The selected self-report measures of QOL, subjective cognition and shared decision making have relevance to both PWE and HOBSCOTCH interventionists, place minimal burden on program participants, offer wide applicability and possess the ability to inform action for continuous improvement. Of further importance, when evaluating the long-term impact of behavioral education programs such as HOBSCOTCH, applied pragmatic measures allow for tracking progress toward population health level objectives and advancing knowledge that brings potential for shaping policy and enhancing clinical practice. Each of these dimensions of health care must be addressed to ensure an evidence based program like HOBSCOTCH is translated from science to sustainable support for PWE who are met with daily cognitive challenges. Future analysis of factors associated with participant response to the intervention in real world delivery and examination of sustained individual-level significant improvements, including improved health and psychosocial outcomes and measures of health care utilization, will allow for enhanced practice guidelines relative to the role of self-management interventions in epilepsy to emerge; and can lend support for policy change that supports reimbursement for the systematized delivery of evidence based ESM in clinical settings.

This report demonstrates how purposeful strategic efforts around education and partnering operationalized via the HOBSCOTCH Institute have facilitated clinician, community and participant engagement in ESM through the creation of easily accessible channels for direct referral from local, regional and national partners. Awareness and visibility of the HOBSCOTCH program were enhanced through multidimensional education initiatives with target groups to facilitate scalable public health impact.

Although seizures are the most prominent feature of epilepsy, many patients view cognitive impairment as the most significant disability (25, 56–58). Patient surveys demonstrate that cognitive and behavioral issues are among their highest concerns (59), data that is further supported by studies revealing cognitive function as a significant predictor of lowered quality of life in PWE (60).

Cognitive and behavioral interventions, including HOBSCOTCH, are low-risk adjuncts to standard therapies and have a place in routine clinical care streams and community support settings to help lower the burden of cognitive dysfunction for PWE. Program evaluation and quality improvement efforts to fully explore the potential for broad implementation of HOBSCOTCH are key to ensuring that they are acceptable for integration in routine clinical practice settings and will allow for more robust health systems integration to fill critical gaps in cognitive rehabilitation options for PWE.

### 4.1 Limitations

The current program evaluation shares data from a centralized hub of telehealth ESM delivery. A rigorous infrastructure of training and Coaching support has been developed at the HOBSCOTCH Institute, facilitating a high level of Cognitive Coach competency for program delivery with good fidelity and consistency. Further program evaluation that incorporates intervention delivery in diverse settings with trained Cognitive Coaches working beyond the centralized HOBSCOTCH Institute is required to further advance and ensure access to self-management support targeting QOL and cognition for PWE through broad educational delivery of the HOBSCOTCH program outside of clinical trials. The absence of objective cognition data in this cohort is a limitation, however, prior randomized controlled data demonstrating patient benefit post-HOBSCOTCH (30–33) have included objective measures and outcomes, and the intent of the current study was to examine real-world implementation with intentionally low participant burden voluntary self-report measures. Given the real-world collection of voluntary survey data and with minimizing survey burden for participants in an adjunct to care psychoeducation program, data limitations include that not all participants completed all requested measures and epilepsy characteristics data are limited to those self-reported variables presented (GASE, seizure control, presence of seizure in the past 30 days or 12 months, self-reported seizure treatment category). Extending the reach of future survey collection tools to include more extensive self-report data of seizure variables such as number and type of antiseizure medications and adherence may lend further insights for interpreting data, recognizing that real-world self-report data will inherently reflect the accuracy, precision and recall of the reporters.

## 5 Conclusion

Increasing healthcare and social services systems capacity to provide equitable access to quality epilepsy care and community support including the integration of the telehealth-deliverable HOBSCOTCH program as a best practice standard is crucial for PWE experiencing related cognitive comorbidity. HOBSCOTCH carries a great promise to improve the lives of PWE who face cognitive challenges in their home, work, school and social environments. Working to enhance referral pathways and collaboration amongst clinical and community settings where PWE receive medical care and resource support will be vital to ensure progress in QOL and cognitive outcomes for those impacted by epilepsy.

## Data Availability

The raw data supporting the conclusions of this article will be made available by the authors, without undue reservation.

## Footnotes

1 See www.dartmouth-hitchcock.org/hobscotch-institute

2 See www.dartmouth-hitchcock.org/hobscotch-institute

3 See https://managingepilepsywell.org/
